# Revisiting the Value of Admission Cardiotocography in Term Pregnancies: An Updated Systematic Review and Meta‐Analysis

**DOI:** 10.1111/1471-0528.70047

**Published:** 2025-10-11

**Authors:** Mariana Tome, Aimée A. K. Lovers, Lawrence Impey, Jane E. Hirst, Antoniya Georgieva

**Affiliations:** ^1^ Oxford Labour Monitoring, Nuffield Department of Women's & Reproductive Health University of Oxford Oxford UK; ^2^ Department of Obstetrics and Gynaecology, Amsterdam UMC Amsterdam the Netherlands; ^3^ The George Institute for Global Health, School of Public Health Imperial College London London UK

**Keywords:** admission cardiotocography, fetal monitoring, intermittent auscultation, labour, pregnancy, screening test, systematic review

## Abstract

**Background:**

Admission cardiotocography (CTG) remains widely used to assess the fetal condition at the onset of labour in low‐risk pregnancies, despite international guidelines recommending against its routine use and advocating intermittent auscultation (IA) instead.

**Objective:**

To evaluate the effect of admission CTG compared with IA upon admission on maternal and neonatal outcomes in low‐risk term pregnancies.

**Search Strategy:**

PubMed and the Cochrane Library were searched from inception to April 2025.

**Selection Criteria:**

Randomised controlled trials (RCTs) comparing admission CTG with IA in low‐risk, term pregnancies with singleton fetuses.

**Data Collection and Analysis:**

Two reviewers independently screened studies, extracted data, and assessed risk of bias using RoB 2.0 from the Cochrane Library. Random‐effects meta‐analysis generated relative risks (RRs), and the quality of the evidence was evaluated using GRADE.

**Main Results:**

Five RCTs (16 341 pregnant women) were included. Admission CTG was associated with significantly higher use of continuous electronic fetal heart rate (FHR) monitoring (RR 1.23, 95% CI: 1.05–1.44, *I* ^2^ = 95%). No significant differences were found for caesarean section (RR 1.09, 95% CI: 0.86–1.37, *I* ^2^ = 49%), fetal blood sampling (RR 1.16, 95% CI: 0.96–1.40, *I* ^2^ = 44%), instrumental deliveries (RR 1.06, 95% CI: 0.90–1.19, *I* ^2^ = 38%), neonatal intensive care unit (NICU) admission (RR 1.07, 95% CI: 0.91–1.27, *I* ^2^ = 0%), or Apgar score < 7 at 5 min (RR 0.97, 95% CI: 0.62–1.54, *I* ^2^ = 13%). Evidence certainty was rated moderate for most outcomes.

**Conclusions:**

Routine admission CTG in low‐risk term pregnancies demonstrated no improvement in maternal or neonatal outcomes. Previous concerns regarding increased caesarean delivery rates appear overstated. These findings support current recommendations favouring IA over routine admission CTG in low‐risk pregnancies. Future research should focus on intermediate‐risk populations, standardised implementation and clinically meaningful outcomes.

**Trial Registration:**

PROSPERO registration number: CRD420251028693

**Abbreviations**
ADCAR: admission cardiotocography
BPM: beats per minute
CI: confidence interval
CTG: cardiotocography
EFM: electronic fetal monitoring
FHR: fetal heart rate
GRADE: Grading of Recommendations, Assessment, Development and Evaluations
IA: intermittent auscultation
LSCS: lower segment caesarean section
NICU: neonatal intensive care unit
NR: not reported
PPI: Patient and Public Involvement
PRISMA: Preferred Reporting Items for Systematic Reviews and Meta‐Analyses
RR: relative risk
RCT: randomised controlled trial
ROB 2.0: revised cochrane risk of Bias tool for randomised trials
SCBU: special care baby unit
SD: standard deviation
UK: United Kingdom

## 1 Introduction

Assessment of fetal health at the onset of labour is a critical component of intrapartum care [1]. The United Kingdom (UK) does not routinely recommend admission CTG for low‐risk pregnancies but limits its use to pregnancies at increased risk for adverse outcomes [2]. In contrast, many countries, for example, the United States, Spain, France and several Eastern European and Asian nations, routinely perform admission CTG for all women [3, 4, 5, 6, 7]. Practices also vary within regions; notably, whilst Sweden and Finland recommend routine admission CTG, other Scandinavian countries such as Denmark and Norway do not [8, 9, 10, 11].

Admission CTG typically involves a 15–20‐min recording that combines fetal heart rate (FHR) and uterine activity. First introduced in 1986, it was intended as a screening tool for low‐risk pregnancies to detect early signs of fetal hypoxia and prompt closer surveillance or timely intervention [12]. Following its introduction, studies published worldwide began trialling the test for other purposes, such as predicting intrapartum complications or identifying fetuses at increased risk of metabolic acidosis at birth [12, 13, 14]. In the following three decades, accumulated evidence from randomised controlled trials (RCTs) and systematic reviews has been relatively consistent: compared with intermittent auscultation (IA), admission CTG does not improve neonatal or maternal outcomes, but is associated with an increase in obstetric interventions. The most recent Cochrane review in 2017 by Devane et al. concluded that admission CTG likely increases the caesarean section rate (RR 1.20, 95% CI 1.00–1.44, *I* ^2^ = 49%) without demonstrable neonatal benefit. Evidence further highlights the limitations of admission CTG as a screening tool, showing low sensitivity and positive predictive value for adverse neonatal outcomes in low‐risk populations [15].

Nonetheless, observational studies suggest that admission CTG can reveal early complications in specific scenarios. Parts et al. found that women who required emergency caesarean section within 1 h of admission frequently had abnormal traces, while Gyllencreutz et al. reported that fetuses with undetected small‐for‐gestational‐age status were more likely to show abnormal admission CTG, and that this combination was strongly associated with worse neonatal outcomes [16, 17]. Although both studies included mixed‐risk populations, they point to a possible but unconfirmed role for admission CTG in detecting rare, clinically important conditions.

Accordingly, international guidelines, including those from the World Health Organization and the UK National Institute for Health and Care Excellence (NICE), recommend against routine admission CTG in low‐risk pregnancies, citing no proven benefit and a risk of unnecessary intervention [18, 19].

Despite the consistent evidence base, admission CTG remains in widespread use, even in settings where it is not recommended. We have therefore conducted an updated systematic review and meta‐analysis to evaluate the effect of admission CTG compared with IA at admission on maternal and neonatal outcomes in low‐risk term pregnancies. Our aim was to strengthen the evidence base with the most recent high‐quality trial data, synthesise the consistent findings and inform recommendations for practice and future research.

## 2 Methods

This systematic review and meta‐analysis was conducted in accordance with the PRISMA 2020 guidelines [20]. The completed PRISMA 2020 checklist is available in Table S1.

### 2.1 Eligibility Criteria

We included RCTs and quasi‐randomised trials comparing admission CTG with IA in low‐risk, term pregnancies with singleton fetuses. No multi‐armed studies were included. Reviews and studies that did not meet these criteria (phase I trials, pilot or feasibility studies without relevant outcome data, abstracts without full‐text manuscripts and observational studies) were not eligible for inclusion. Studies were eligible if they reported on at least one of the following outcomes: adverse neonatal outcomes, mode of delivery, intrapartum interventions and abnormal CTG rate (Table S2).

Admission CTG was defined as a brief recording of FHR and uterine activity performed upon admission to the labour ward or assessment unit. IA was defined as intermittent monitoring using a Pinard stethoscope or Doppler device, without continuous waveform display.

Studies evaluating antenatal CTG, continuous CTG used during labour (rather than as an admission test), other admission tests apart from CTG (e.g., ultrasound), fetal movement counting, medication‐related interventions or induction‐of‐labour interventions were not eligible.

### 2.2 Search Strategy and Study Selection

We searched PubMed and the Cochrane Library from inception to April 2025 using keywords and MeSH terms related to ‘cardiotocography,’ ‘fetal monitoring’ and ‘admission’ (Table S3). No filters were applied to restrict the search to RCTs, to maximise sensitivity; eligibility by study design was determined during title/abstract and full‐text screening. Reference lists of relevant systematic reviews (including Devane et al. [1]) were also screened to identify additional eligible trials, but no further trials were identified. No language restrictions were applied. Two reviewers (M.T. and A.L.) independently screened titles and abstracts, assessed full texts for eligibility, and extracted data using a standardised form. Discrepancies were resolved through discussion and reviewed by the senior investigator (A.G.). Systematic reviews and other study designs that did not meet the eligibility criteria (e.g., narrative reviews) were excluded at the full‐text screening stage.

### 2.3 Quality Assessment

The methodological quality of included studies was independently assessed using the Critical Appraisal Skills Programme (CASP) checklist and the Oxford Centre for Evidence‐Based Medicine tool [21, 22].

### 2.4 Risk of Bias Assessment

The risk of bias in each included RCT was assessed using the Revised Cochrane Risk of Bias tool for randomised trials (RoB 2.0) [23]. Two reviewers (M.T. and A.L.) independently evaluated each domain, with discrepancies resolved by consensus. Publication bias was not formally assessed due to the limited number of studies per outcome (< 10). Risk of bias and heterogeneity in study design were considered narratively in the interpretation of findings.

### 2.5 Certainty of Evidence

The Grading of Recommendations Assessment, Development and Evaluation (GRADE) approach was used to assess the certainty of evidence for each outcome, considering risk of bias, inconsistency, indirectness and imprecision [24]. Outcomes were rated as high, moderate, low, or very low certainty. Summary of Findings tables were generated to present key outcomes and corresponding GRADE assessments.

### 2.6 Data Synthesis and Statistical Analysis

Categorical variables were summarised as frequencies and percentages, and continuous variables as means and standard deviations, either as reported or calculated from available data. Meta‐analysis was performed using a random‐effects model (DerSimonian and Laird) with inverse‐variance weighting [25]. The RRs with 95% CIs were calculated for outcomes of interest. A continuity correction of 0.5 was applied where appropriate.

Data were extracted directly from the published trial reports. Where discrepancies existed with the latest Cochrane review by Devane et al., we cross‐checked both sources and used corrected data reported by Devane et al. from the original trial authors (Mires et al., Cheyne et al., Impey et al.) to ensure consistency and accuracy.

Heterogeneity was assessed using Cochran's *Q* test and quantified via the *I* ^2^ statistic, with thresholds of 25%, 50% and 75% representing low, moderate and high heterogeneity, respectively. All analyses were performed using R (version 4.0.3) with the *metafor*, *meta,* and *metabin* packages.

## 3 Results

**FIGURE 1:**
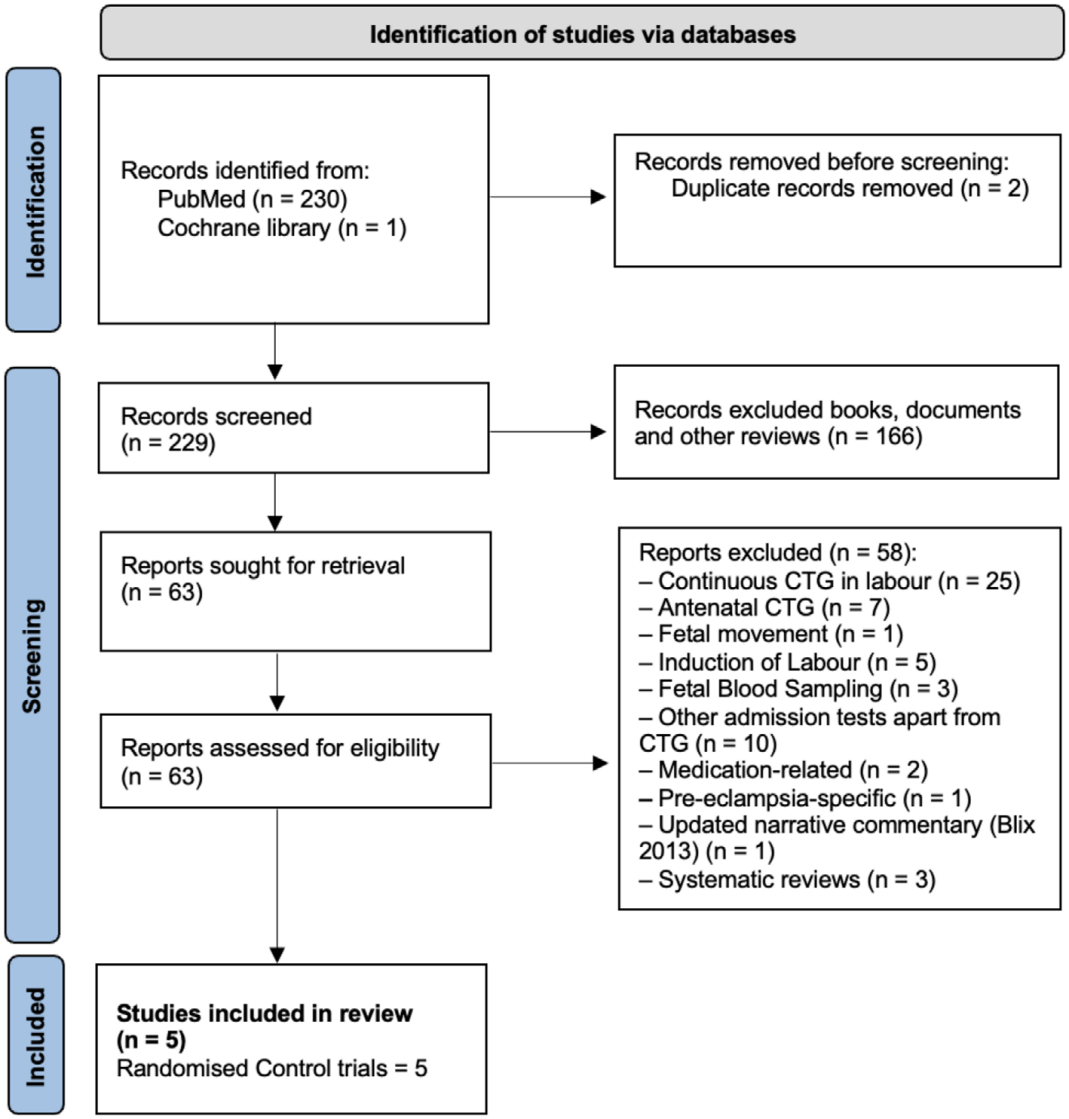
PRISMA flow diagram.

A total of 231 publications were identified (Figure 1). After removing duplicates (*n* = 2), 229 records were screened at title and abstract level, of which 166 were excluded. Sixty‐three full‐text articles were assessed for eligibility, and 58 were excluded for reasons detailed in Figure 1. Five RCTs met the inclusion criteria and were included in the review. Study characteristics, including primary and secondary outcomes, are summarised in Table 1 [26, 27, 28, 29, 30].

**TABLE 1:** Summary of study characteristics of RCTs included in the systematic review and meta‐analysis.

	Mires et al. [26] (2001)	Cheyne et al. [27] (2003)	Impey et al. [28] (2003)	Mitchell et al. [29] (2008)	Smith et al. [30] (2019)
Country/Setting	Scotland; Teaching hospital	Scotland; Midwives‐led birth unit in teaching hospital	Ireland; Teaching hospital	England; Regional hospital	Ireland; Two tertiary birth centres, one regional birth centre
Participant recruitment	Third trimester	Labour ward with possible labour onset.	Labour ward with possible labour onset.	Labour ward with possible labour onset	Third trimester
Inclusion criteria	Low‐risk pregnancy	Low‐risk pregnancy	Low‐risk pregnancy	Low‐risk pregnancy.	Low‐risk pregnancy
Key exclusion criteria	Multiple pregnancies, Previous caesarean section; Breech position; Pre‐eclampsia	Presence of risk factors (not specified)	Multiple pregnancies; suspicion of antenatal fetal compromise; adverse obstetric	Multiple pregnancy; labour induction; suspected fetal medical complication; poor obstetric history (e.g., history of stillbirth)	Multiple pregnancies; Preterm and post‐term labour post‐term Previous Caesarean section
Randomisation	After formal diagnosis of labour onset	After formal diagnosis of labour onset	After formal diagnosis of labour onset	After formal diagnosis of labour onset	Before formal diagnosis of labour onset (i.e., possible labour onset)
Sample size	3752	334	8628	582	3045
Details admission CTG	20 min after labour diagnosis; abnormal definition not specified	20 min after labour diagnosis; abnormal definition not specified	20 min after labour diagnosis. Definition of abnormal: not normal FHR (i.e., baseline 110–160 bpm, variability < 5 bpm, ≥ 1 acceleration, no decelerations)	Minimum 15 min after labour diagnosis until obstetric team found normal trace by NICE 2001 guidelines; abnormal not specified	20 min after labour diagnosis Definition of abnormal: not normal FHR (i.e., baseline 110–160 bpm, variability > 10 bpm, ≥ 2 acceleration, no decelerations)
Details IA	After formal diagnosis of labour; IA with hand‐held doppler during and immediately after at least one contraction; duration not specified	After formal diagnosis of labour; IA with hand‐held doppler during a contraction and for ≥ 60 s immediately after	After formal diagnosis of labour; IA instrument not specified; ≥ 1 min after a contraction every 15 min in first stage and every 5 min in second stage. This was done after early amniotomy on diagnosis of labour. Escalation to EFM if decelerations, tachycardia, meconium/bloodstained liquor, maternal temperature ≥ 38°C or labour > 8 h	After formal diagnosis of labour; IA with Pinard or Doppler; 1 min continuous auscultation after a contraction, every 15 min in first stage and every 5 min in second stage	Before formal diagnosis of labour; IA with pinard or hand‐held doppler for ≥ 60 s after a contraction; repeated every 15 min in first stage and every 5 min in second stage. Conversion to CTG if baseline FHR < 110 bpm or > 160 bpm, decelerations, additional risk factors or clinician concern
Primary outcome	Metabolic acidosis (arterial umbilical cord) pH < 7.20 with base deficit of > 8.0 mmol/L	Continuous CTG^b^; any additional CTG after admission test	Composite: perinatal mortality and morbidity^c^	Operative delivery^a^ rate	Caesarean section rate
Key secondary outcomes	Apgar < 7 at 5 min, resuscitation, NICU admission, fetal scalp sampling, epidural, operative delivery^a^, caesarean section	Apgar < 7 at 5 min, NICU admission, fetal scalp sampling, augmentation, epidural	NICU admission, cord pH, Apgar < 7 at 5 min, continuous CTG, fetal blood sampling, caesarean section	Stillbirth, neonatal death, augmentation, Apgar < 7 at 5 min, NICU admission	Continuous CTG, fetal blood sampling, augmentation, epidural, cord pH < 7.0, Apgar scores, NICU admission
Analysis	Intention‐to‐treat with subgroup analysis^d^	Intention‐to‐treat	Intention‐to‐treat with subgroup analysis^e^	Intention‐to‐treat	Intention‐to‐treat with subgroup analysis^f^

Abbreviations: bpm, beats per minute; CTG, cardiotocography; EFM, electronic fetal monitoring; FHR, Fetal Heart Rate; IA, intermittent auscultation; NICU, neonatal intensive care unit.

^a^ Operative delivery included instrumental vaginal delivery (forceps and ventouse) and caesarean delivery.

^b^ Continuous CTG defined as the use of CTG for ≥ 75% of the time in labour.

^c^ Composite outcome: intrapartum stillbirth; neonatal death within 7 days; NICU admission in addition to arterial umbilical cord pH < 7.05, neonatal seizures; hypotonia > 4 h, resuscitation, inotropic support, renal failure and/or meconium aspiration syndrome.

^d^ Subgroup analysis excluded pregnant women who became ineligible after randomisation (i.e., developed risk factors) based on the following reasons (*n* = 1384): antepartum haemorrhage; raised blood pressure; suspected small for gestational age; preterm labour; gestational diabetes; fetal anatomy; reduced fetal movements and suspected fetal compromise; meconium‐stained amniotic fluid; intrauterine death; persistent breech; pre‐labour ruptured membranes; induction of labour; baby born before arrival at hospital; elective caesarean section; women who withdrew from trial.

^e^ Subgroup analysis excluded pregnant women who had preterm deliveries (*n* = 63), who had labour lasting more than 5 h (*n* = *not specified*), and who did not receive obstetric care that warranted continuous CTG monitoring (i.e., epidural analgesia or labour augmentation with oxytocin; *n* = 3038).

^f^ Subgroup analysis for the primary outcome that excluded pregnant women who became ineligible post‐randomisation (i.e., discharged home for not being in established labour; *n* = 476), and subgroup analysis for both primary and secondary outcome adjusting for maternal age, parity and birth unit.

The five RCTs were conducted between 2001 and 2019 in various maternity settings across the UK and Ireland, including teaching hospitals, midwife‐led units, and regional hospitals (Table 1). Sample sizes ranged from 334 to 8628 participants. Only two studies met their precalculated sample size, and none were adequately powered to detect rare adverse outcomes, such as perinatal severe morbidity or mortality [26, 28]. Smith et al. conducted the only multicentre‐study [30].

Detailed characteristics of participants across the five included trials, including maternal age, parity, gestational age, and birth weight, are presented in Table S4. No details regarding stages of labour or cervical dilation were reported.

### 3.1 Quality of the Evidence

Using the revised Cochrane risk‐of‐bias tool (RoB 2.0), all five RCTs were at low risk of bias for randomisation and outcome completeness, with ‘some concerns’ in at least one domain, most commonly regarding outcome measurement and selection of the reported result (Table 2).

**TABLE 2:** Risk of bias 2 summary table for the five RCTs included in the systematic review.

Study	Randomisation process	Deviations from intended interventions	Missing outcome data	Measurement of the outcome	Selection of the reported result	Overall risk of bias
Mitchell et al. [29] (2008)	Low	Some concerns	Low	Some concerns	Some concerns	Some concerns
Smith et al. [30] (2019)	Low	Low	Low	Some concerns	Some concerns	Some concerns
Mires et al. [26] (2001)	Some concerns	Some concerns	Low	Some concerns	Some concerns	Some concerns
Impey et al. [28] (2003)	Low	Low	Low	Low	Some concerns	Some concerns
Cheyne et al. [27] (2003)	Low	Low	Low	Low	Some concerns	Some concerns

Most key outcomes, including caesarean section, continuous fetal monitoring, and NICU admissions, were rated as moderate‐certainty evidence (GRADE), due to a lack of blinding and unclear reporting plans across all studies (Table 3). For less frequent outcomes, such as hypoxic‐ischaemic encephalopathy, evidence was downgraded to low certainty due to both imprecision (wide confidence intervals crossing the line of no effect) and risk of bias. None of the assessed outcomes achieved high‐certainty ratings.

**TABLE 3:** GRADE summary of the outcome measures.

Outcome	No. of participants (studies)	Effect estimate (95% CI)	Certainty of evidence (GRADE)	Comments
Caesarean section	14 372 (5 RCTs)	RR 1.09 (0.86–1.37)	Moderate	Downgraded one level for risk of bias (lack of blinding in all studies, no pre‐specified analysis plans). Effect estimates no longer significant with inclusion of recent data.
Instrumental vaginal delivery	14 372 (5 RCTs)	RR 1.06 (0.95–1.19)	Moderate	Downgraded one level for risk of bias (lack of blinding may influence decisions for instrumental delivery). Consistent findings across studies.
Neonatal admission to NICU	14 365 (5 RCTs)	RR 1.07 (0.91–1.27)	Moderate	Downgraded one level for risk of bias and imprecision (wide confidence interval includes both potential benefit and harm).
Continuous EFM	13 786 (4 RCTs)	RR 1.23 (1.05–1.44)	Moderate	Downgraded one level for risk of bias. Substantial heterogeneity present (*I* ^2^ = 95%) but consistent direction of effect and statistical significance maintained.
Fetal blood sampling	13 791 (4 RCTs)	RR 1.16 (0.96–1.40)	Moderate	Downgraded one level for risk of bias and inconsistency. Previously significant association no longer significant with inclusion of recent data.
Apgar score < 7 at 5 min	14 358 (5 RCTs)	RR 0.97 (0.62–1.54)	Moderate	Downgraded one level for risk of bias and imprecision. Low event rates limit precision.
Hypoxic‐ischaemic Encephalopathy	5401 (2 RCTs)	RR 0.88 (0.30–2.56)	Low	Downgraded two levels for very serious imprecision (very wide confidence interval, very few events) and risk of bias.

*Note:* GRADE certainty ratings: High certainty—the true effect is likely to be close to the estimated effect; Moderate certainty—the true effect is probably close to the estimate, but it may be substantially different; Low certainty—limited confidence in the effect estimate; the true effect may be substantially different; Very low certainty—very little confidence in the effect estimate; the true effect is likely to be substantially different. All studies were initially rated as high‐certainty evidence (as randomised controlled trials), with downgrades based on identified study limitation.

Abbreviations: CI, confidence interval; EFM, electronic fetal monitoring; GRADE, Grading of Recommendations, Assessment, Development and Evaluations; NICU, neonatal intensive care unit; RCT, randomised controlled trial; RR, risk ratio; SCBU, special care baby unit.

### 3.2 Admission CTG Criteria and Participant Selection

Considerable methodological heterogeneity was observed across studies. Participant recruitment differed, with some studies enrolling women during their pregnancy and others only at labour ward admission. One study included women with ‘possible’ labour onset, whilst others included those with formal labour diagnoses [30].

Definition of admission CTG varied, with most studies requiring a 20‐min recording, but Mitchel et al. accepted a 15‐min trace [29]. The criteria for normal versus abnormal CTG traces were also heterogeneous. Rates of abnormal admission CTG ranged from 21.5% to 32% (Table S5). Moreover, Impey et al. [28] included patients with a higher risk profile (< 1% vaginal breech, 1% preterm births, 8% of pregnancies with previous deliveries by caesarean section), and offered all nulliparous women early active labour management, including early amniotomy.

### 3.3 Delivery Outcomes

**FIGURE 2:**
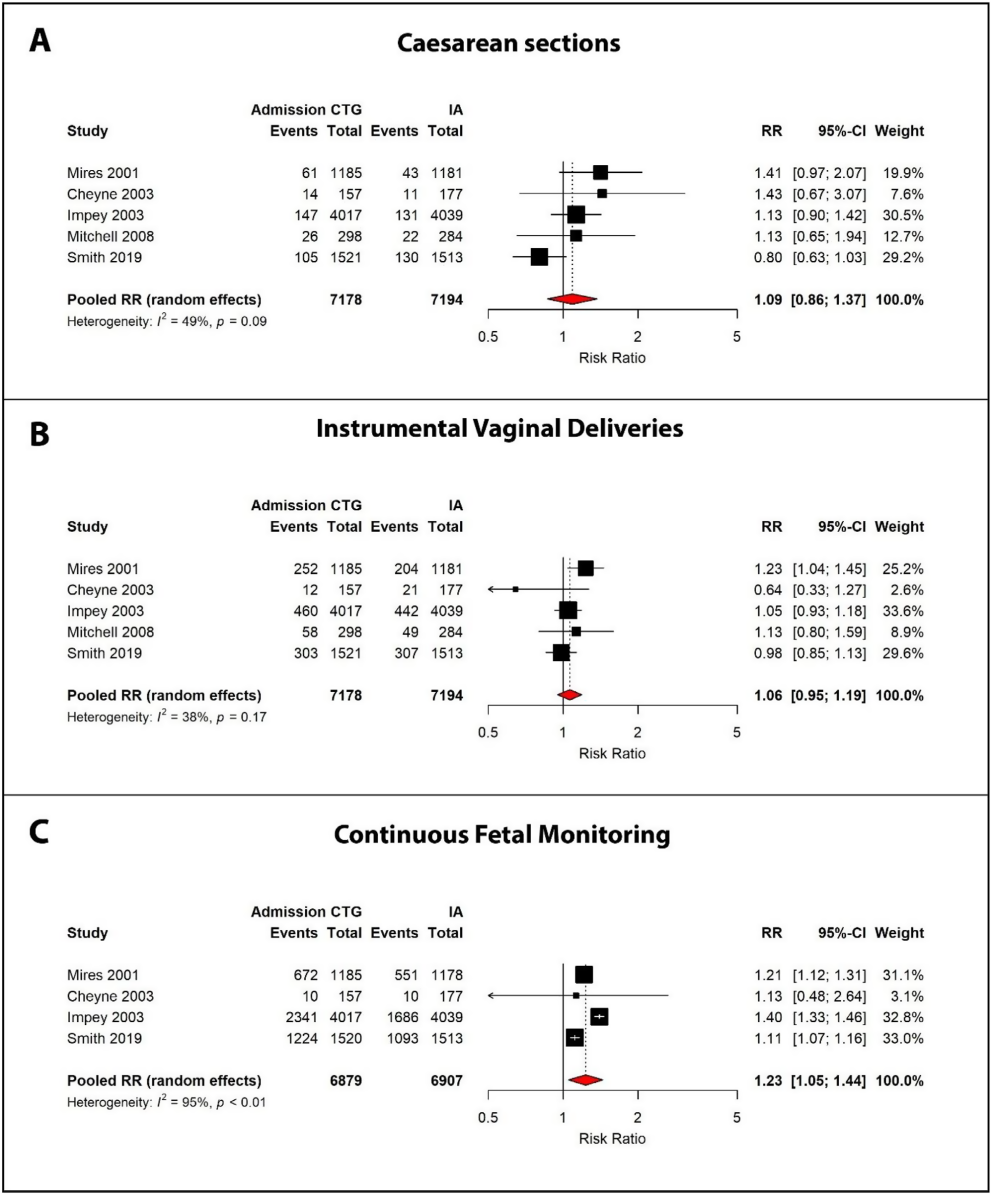
Forest plots comparing mode of delivery outcomes between admission cardiotocography (CTG) and intermittent auscultation (IA) for (A) caesarean sections, (B) instrumental vaginal deliveries and (C) shows the rates of continuous fetal monitoring.

All five RCTs reported on caesarean section and instrumental vaginal delivery outcomes. The rate of caesarean section in the admission CTG cohort ranged from 3.7% to 8.9%, compared with 3.2% to 8.6% in the IA cohort (Table S6) [26, 27, 28, 29, 30]. Caesarean section rates were higher in women undergoing admission CTG monitoring across all studies, except Smith et al. [30] Overall, the pooled caesarean section rate was 4.9% in the CTG group and 4.6% in the IA group, yielding a non‐significant difference (RR 1.09, 95% CI: 0.86–1.37, *I* ^2^ = 49%) (Table 4, Figure 2A).

**TABLE 4:** Summary of the pooled estimates for the pre‐specified outcomes.

Outcome groups	Outcome measures	Studies (*N*)	Participants (*N*)	Updated relative risk (95% CI)	Previous^b^ relative risk (95% CI)	Change	Evidence^c^ updated
Mode of Delivery Outcomes	Incidence of Caesarean section	5	14 372	1.09 (0.86–1.37)	1.20 (1.00–1.44)	No longer significant	Yes
	Instrumental Vaginal Deliveries	5	14 372	1.06 (0.95–1.19)	1.10 (0.95–1.27)	No Change	Yes
Intrapartum Management	Continuous Fetal Monitoring	4	13 786	1.23 (1.05–1.44)	1.30 (1.14–1.48)	No Change	Yes
	Fetal Blood Sampling	4	13 791	1.16 (0.96–1.40)	1.28 (1.13–1.45)	No longer significant	Yes
	Amniotomy	3	5728	1.05 (0.98–1.13)	1.04 (0.97–1.12)	No Change	Yes
	Epidural	4	13 795	1.03 (0.92–1.16)	1.11 (0.87–1.41)	No Change	Yes
	Oxytocin	5	14 372	1.03 (0.96–1.10)	1.05 (0.95–1.17)	No Change	Yes
Fetal and Neonatal Outcomes	NICU admissions	5	14 365	1.07 (0.91–1.27)	1.03 (0.85–1.24)	No Change	Yes
	Apgar score < 7^a^	5	14 358	0.97 (0.62–1.54)	1.00 (0.54–1.85)	No Change	Yes
	Fetal and Neonatal Deaths	5	14 373	1.02 (0.33–3.14)	1.01 (0.30–3.47)	No Change	Yes
	Hypoxic encephalopathy	2	5401	0.88 (0.30–2.56)	1.19 (0.37–3.90)	No Change	Yes

Abbreviations: CI, confidence interval; NICU, neonatal intensive care unit; RR, relative risk.

^a^ At or after 5 min.

^b^ Relative risks and 95% confidence intervals from the previous meta‐analysis (Declane et al. [1]) are shown for comparison. ‘Change’ refers to a shift in the statistical interpretation (e.g., from non‐significant to significant) or a major change in effect estimate.

^c^ Updated meta‐analysis includes a new study.

In most studies, instrumental vaginal delivery rates were higher than caesarean section rates, with the exception of Cheyne et al. [27] Instrumental delivery occurred in 15.1% of women in the CTG group and 14.2% in the IA group, resulting in a pooled RR of 1.06 (95% CI: 0.90–1.19, *I* ^2^ = 38%) (Table 4, Figure 2B).

### 3.4 Intrapartum Management

**FIGURE 3:**
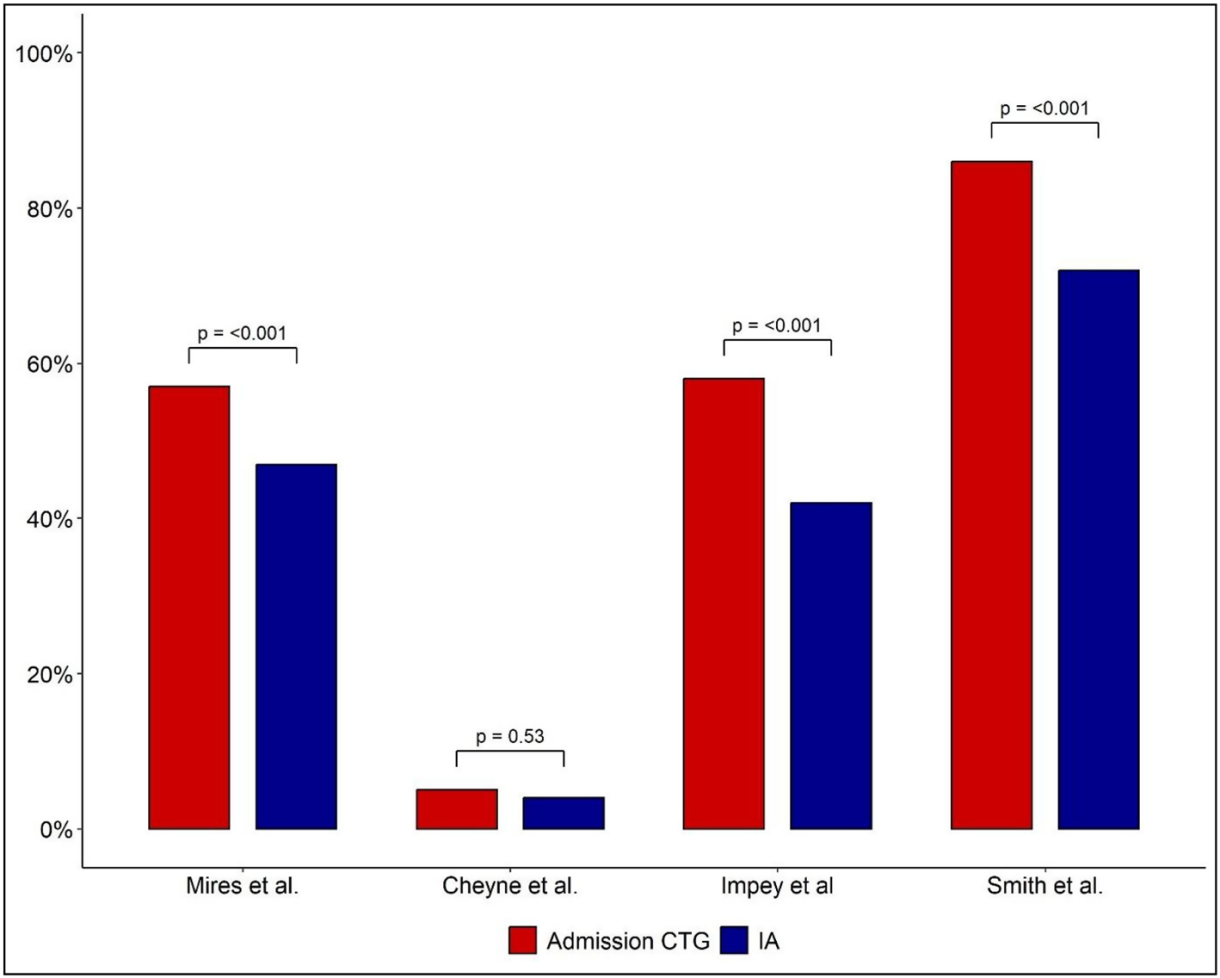
Comparison of continuous CTG use between admission CTG and IA across the four randomised controlled trials. Mitchell et al. did not report the rates of continuous CTG. CTG, cardiotocography; IA, intermittent auscultation.

Admission CTG was associated with a trend towards increased intrapartum interventions (Figure 2C). Continuous fetal monitoring showed the most consistent effect across studies, with all four studies reporting increased rates in admission CTG groups. Impey et al. demonstrated the largest relative difference (58.3% vs. 41.7%; RR 1.40), whereas Smith et al. reported more modest differences (80.5% vs. 72.2%; RR 1.11) (Figure 3) [28, 30]. This resulted in a significantly higher rate of continuous monitoring with admission CTG (RR 1.23, 95% CI: 1.05–1.44, *I* ^2^ = 95%), despite considerable heterogeneity (*I* ^2^ = 95%).

There was a considerable difference in fetal blood sampling, with only Impey et al. reporting a statistically significant increase in the admission CTG group compared to IA (11% and 8%, respectively) [28]. However, the overall pooled results showed a trend towards higher rates in the admission CTG cohort but no longer significant (RR 1.16, 95% CI: 0.96–1.40, *I* ^2^ = 0%). Similarly, no significant differences between admission CTG and IA were found for amniotomy, epidural use and oxytocin administration (Figure S1).

### 3.5 Fetal and Neonatal Outcomes

**FIGURE 4:**
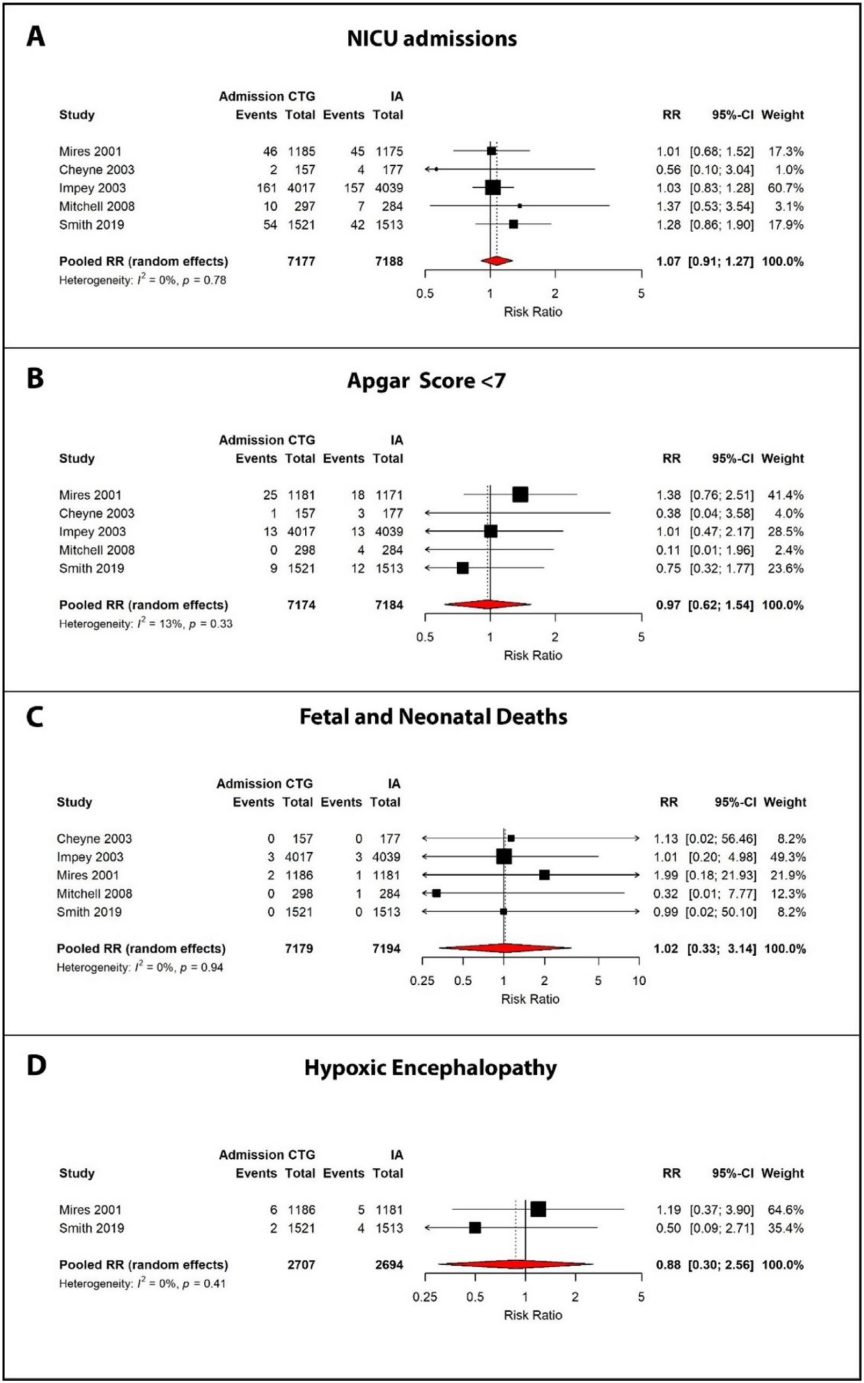
Forest plots comparing fetal and neonatal outcomes between admission cardiotocography (CTG) and intermittent auscultation (IA). (A) Neonatal intensive care unit admissions, (B) Apgar score < 7, (C) Fetal and neonatal deaths and (D) Hypoxic encephalopathy.

All studies reported neonatal outcomes including NICU admission, Apgar scores < 7 at 5 min, and fetal and neonatal mortality. Admission CTG was associated with a non‐significant increase in NICU admissions (RR 1.07, 95% CI: 0.91–1.27, *I* ^2^ = 0%), although the absolute event numbers were low, particularly in Cheyne et al. (Figure 4A) [27].

Rates of low Apgar scores (< 7 at 5 min) were low across all studies, with no significant differences between groups (RR 0.97, 95% CI: 0.62–1.54, *I* ^2^ = 13%) (Figure 4B). Despite enrolling over 8000 women, Impey et al. [28] reported only a modest number of cases with low Apgar scores.

For the critical outcome of perinatal mortality, a total of 10 deaths were reported across the five trials. Impey et al. reported six deaths, including five in babies with major congenital anomalies, whilst Mires et al. noted three intrauterine deaths diagnosed post‐randomisation but prior to the onset of labour [26, 28]. Finally, Mitchell et al. reported one death in the IA group (Figure 4C) [29].

Hypoxic‐ischaemic encephalopathy was reported in only two of the five trials. Smith et al., in the most recent study, reported a non‐significant reduction in risk (RR 0.50), in contrast to Mires et al., which found a higher rate in the admission CTG group (RR 1.19) [26, 30]. However, the overall estimate remained non‐significant due to the small number of events (Figure 4D).

**FIGURE 5:**
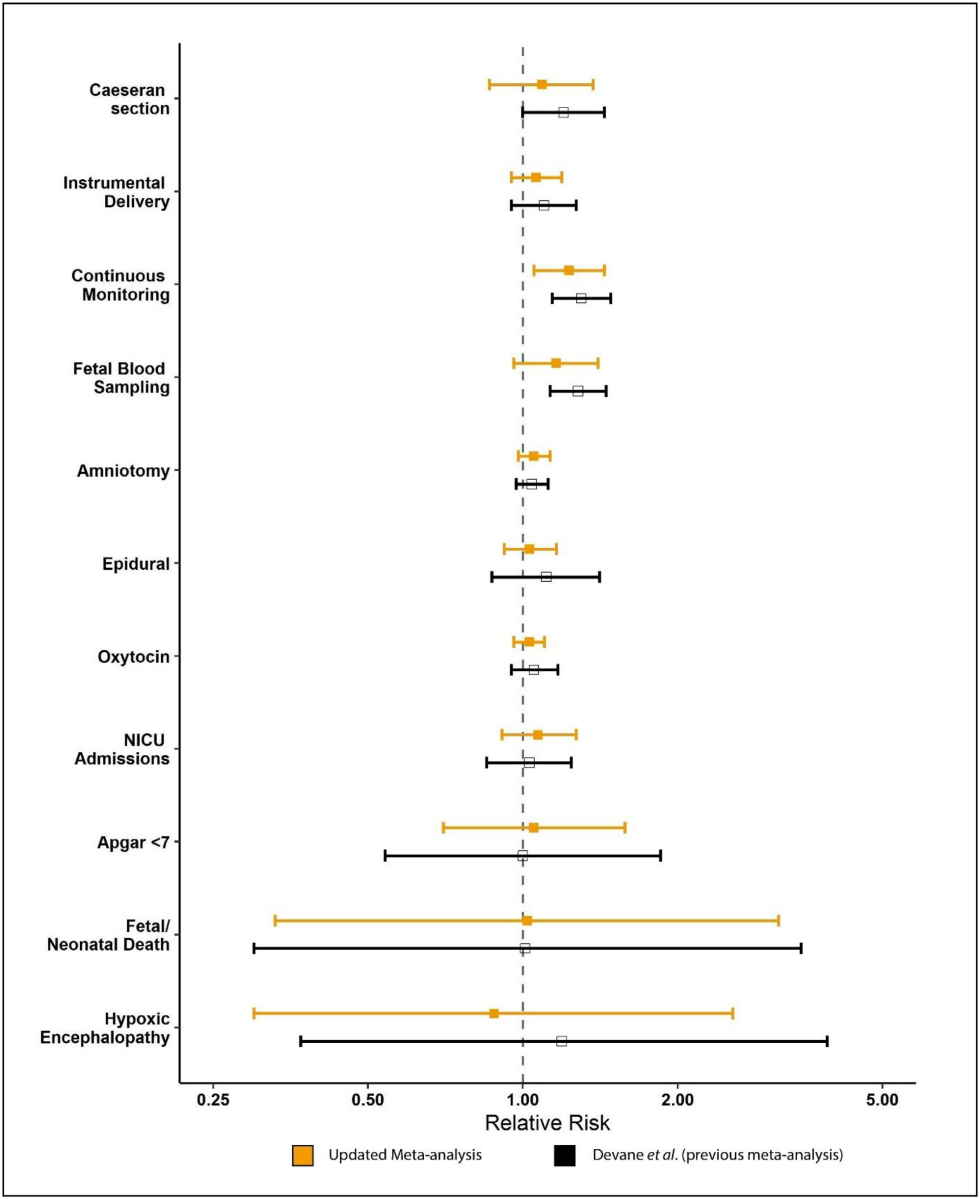
Forest plots comparing our findings with the previous systematic review and meta‐analysis (Devane et al. [1]).

Comparison of the point estimates with the previous Cochrane review (Devane et al.), which did not include Smith et al., is depicted in Figure 5 [1, 30].

## 4 Discussion

### 4.1 Main Findings

This updated systematic review and meta‐analysis, incorporating over 16 000 low‐risk term pregnancies across five RCTs, provides the most comprehensive evidence to date on admission CTG.

The inclusion of Smith et al. [30] expands the evidence base by more than 3000 additional women, increasing the total sample size from 13 296 to 16 341 women (a 23% increase) and offers greater precision in our understanding of admission CTG. Our analysis found no significant association between admission CTG and caesarean section rates (RR 1.09, 95% CI: 0.86–1.37, *I* ^2^ = 49%), contrasting with the earlier Cochrane review which suggested a probable increase (RR 1.20, 95% CI: 1.00–1.42). While the direction of evidence is unchanged, this updated synthesis strengthens confidence that admission CTG does not increase caesarean risk. Importantly, our findings confirm that admission CTG does not improve maternal or neonatal outcomes and therefore should not be recommended for routine use in low‐risk women.

Despite this shift in caesarean section risk, admission CTG continues to be associated with significantly increased continuous electronic fetal monitoring during labour (RR 1.23, 95% CI: 1.05–1.44, *I* ^2^ = 95%). In absolute terms, continuous monitoring was implemented in 57.3% of women in admission CTG groups compared to 46.7% in IA groups, representing an absolute increase of 10.6 percentage points. This increased monitoring occurred despite considerable heterogeneity in baseline monitoring rates across studies (*I* ^2^ = 95%), ranging from just 5% in Cheyne et al. to 80.5% in the more contemporary cohort Smith et al. [27, 30].

Our GRADE assessment showing moderate‐certainty evidence for key outcomes strengthens confidence in these findings and supports current recommendation against routine use of admission CTG in low‐risk pregnancies. However, the rationale should be refined; guidelines and risk–benefit discussions with women should emphasise the lack of maternal and fetal benefits rather than just citing an increase in caesarean rates. Taken together, these findings provide reassurance that admission CTG does not increase caesarean rates but also reinforce that it offers no maternal or neonatal benefit, supporting its continued non‐recommendation in low‐risk women.

### 4.2 Clinical Relevance and Practice Implications

Despite clear guideline recommendations favouring IA, admission CTG remains widely used in high‐income settings [3, 4, 5, 6, 7]. This implementation gap reflects multiple factors beyond the simple evidence‐practice disconnect. Clinicians often cite medicolegal concerns, with admission CTG viewed as ‘defensive practice’ that documents fetal health at a specific time‐point [31, 32]. Institutional culture also plays a significant role, with established routines proving resistant to change despite evolving evidence [33, 34]. Moreover, the relatively limited training in IA skills compared to electronic monitoring interpretation may contribute to clinician preference for technology‐based approaches [35, 36]. Finally, there appears to be a persistent belief, not supported by current evidence, that admission CTG provides added value in risk stratification that is not captured by the existing literature [27, 31, 32, 35].

Understanding these barriers is essential to developing effective implementation strategies that bridge the gap between evidence and practice, ensuring that intermittent auscultation or admission CTG is used appropriately for the right woman, in the right context, at the right time.

Our findings suggest several practical considerations for clinicians and clinical units. First, maternity services should develop robust risk stratification tools with clearly defined criteria to identify women who can safely be monitored with IA alone. This includes reaching consensus on the threshold and weighting of specific risk factors across the spectrum of clinical risk, to avoid unnecessary use of admission CTG. Second, when used, admission CTG should be limited to the standardised 20 min duration, as longer traces (> 1 h) are linked to higher interventions [29]. Third, maternal units should prevent a cascade of interventions by implementing standardised interpretation with objective classification systems, and by critically evaluating whether continuous monitoring following the initial 20‐min admission CTG is warranted as it may trigger unjustified escalation of care [32, 37, 38]. Last, shared decision‐making should be based on the available evidence that suggests a lack of benefits in maternal and fetal outcomes when using admission CTG in low‐risk pregnancies.

### 4.3 Research and Policy Implications

While current evidence does not support routine use of admission CTG in low‐risk pregnancies, its persistent use despite guideline recommendations suggests unresolved clinical questions and implementation challenges. The available trials differ substantially in design, population and implementation, limiting definitive conclusions. Given the heterogeneity across RCTs, future research should clarify where, if anywhere, admission CTG might hold value. We highlight five priorities:

1. Refining the Target Population: Admission CTG may detect pathology in high‐risk pregnancies, but leads to false positives in low‐risk groups. It may hold value for intermediate‐risk pregnancies, which remain understudied. Most trials applied broad low‐risk criteria, yet clinical presentations often fall along a risk continuum rather than a binary classification. Impey et al. [28], the largest RCT, excluded women with meconium‐stained liquor through early amniotomy, lowering baseline risk and complicating comparisons for low‐risk women when liquor colour is unknown. Improved antenatal risk stratification may help identify subgroups that could benefit from early intrapartum assessment.
2. Timing and Purpose: Studies varied in whether admission CTG was performed before or after confirmation of labour onset. Protocols should specify whether the test is intended as a screening tool for pre‐existing injury or a physiological assessment of fetal reserve, as this distinction affects implementation and outcomes.
3. Admission CTG Duration: Most RCTs implemented a brief (~20‐min) recording, though direct comparisons are lacking. Mitchell et al. [29] reported CTGs > 1 h were associated with increased operative delivery. At term, fetuses alternate between sleep and wake states in 20–50‐min cycles [39, 40]. Further studies should confirm whether 20 min is sufficient to detect non‐reactive traces, particularly during quiet sleep.
4. Definition of Abnormal CTG: Only two studies clearly defined abnormal patterns, contributing to heterogeneity. Objective, physiology‐based classification systems may improve consistency [41, 42]. Importantly, certain fetal heart rate characteristics often associated with chronic hypoxia or pre‐existing injury (such as reduced variability, absence of cycling and presence of shallow decelerations) may be difficult to identify with intermittent auscultation alone [16, 43, 44, 45, 46, 47]. However, it should be mentioned that the ability of IA to detect these features, compared with CTG, has not been formally studied. Novel approaches, including computerised analysis and machine learning, could reduce subjectivity and warrant evaluation.
5. Strengthening the Evidence Base for IA: Further research is needed to expand the evidence base for IA, the recommended method for low‐risk women. Key uncertainties remain regarding optimal practice: Pinard versus handheld Doppler, duration and timing of auscultation, and definitions of normal versus abnormal FHR. Trials evaluating structured IA training and adherence to guideline‐recommended monitoring could show whether high‐quality IA improves outcomes or reduces reliance on admission CTG.
6. Outcome Measures: Rare outcomes, such as perinatal mortality and hypoxic encephalopathy, are too infrequent for moderate‐sized trials [30]. Future studies should evaluate more frequent outcomes (e.g., reduction in emergency caesarean sections, predictive accuracy of admission CTG for later FHR abnormalities). Composite outcomes should be developed specifically for admission CTG evaluation.
7. Future Trials: Adaptive or pragmatic designs, including cluster‐randomised or stepped‐wedge trials, may be more feasible than traditional RCTs given the widespread use of admission CTG in many settings.

### 4.4 Patient Experience and Values

Maternal experience is often overlooked in debates on fetal monitoring. Limited research suggests admission CTG may heighten anxiety, restrict mobility, and medicalise early labour [48, 49]. Our PPI panel echoed these concerns, emphasising autonomy and dignity in monitoring choices while highlighting the frequent lack of consideration for women's views and feelings in decision‐making around monitoring practices. For some women, however, CTG provides reassurance, safety, and the sense of being closely observed by healthcare providers [50, 51]. Future research should incorporate patient‐reported measures and preference‐based outcomes. Maternity services must ensure women receive clear information on the purpose, benefits and limitations of monitoring options, supporting shared decision‐making that balances safety with experience.

### 4.5 Strengths and Limitations

This review includes all five RCTs published to date evaluating admission CTG in high‐income settings, with over 16 341 participants. Inclusion of the Smith et al. trial strengthens the generalisability and contemporary relevance of our findings. Our comprehensive appraisal of study design and implementation adds practical insights for policy and clinical practice. Our GRADE assessment provides a transparent evaluation of evidence certainty, highlighting that whilst our conclusions are supported by moderate‐certainty evidence for most outcomes, methodological issues prevent higher certainty ratings. The consistency of findings across outcomes, despite these limitations, strengthens our overall conclusions.

However, methodological heterogeneity, limited power to detect rare outcomes, and evolving obstetric practices over the study period constrain interpretation. Differences in CTG interpretation criteria, patient selection and intervention protocols limit comparability and generalisability. Moreover, none of the included studies were blinded, which may have influenced clinical decision‐making. These limitations underscore the need for standardised approaches in future research, whilst simultaneously supporting the robustness of our main finding that admission CTG increases interventions without improving outcomes.

Importantly, our meta‐analysis provides strong evidence against the routine use of admission CTG in low‐risk pregnancies based on currently available RCT data. Primarily, with few cases of hyoxia encephalopathy and low certainty of evidence on this outcome, we cannot entirely exclude some potential benefit that could arise either from early identification of compromised fetuses or targeted prevention of intrapartum harm. Our findings only apply where an admission CTG is compared with IA in low‐risk populations. Furthermore, the largest trial specifically excluded women for whom the colour of the amniotic fluid was unknown. The continuing divergence of opinion in this field likely reflects the fact that different stakeholders may be using CTG to address different clinical questions, including enabling more rapid fetal assessment, not all of which have been adequately studied through RCTs.

## 5 Conclusion

Our updated review of five RCTs with over 16 000 women with low‐risk term pregnancies, demonstrates that routine admission CTG does not improve neonatal or maternal outcomes. The addition of Smith et al. [30] in this updated meta‐analysis increased the total sample size and reduced the relative risk for caesarean section from 20% to 9%, which is no longer statistically significant. The perception of increased caesarean section had been a central argument against routine use of admission CTG; however, with updated evidence, this association appears weaker than previously suggested. Nonetheless, the absence of demonstrable maternal or neonatal benefit remains a compelling reason to prioritise IA for low‐risk women.

For maternal units employing admission CTG, standardised protocols (20 min), interpretation criteria, and clear management pathways are essential to avoid an unwarranted cascade of interventions. Targeted studies examining specific contexts where admission CTG might offer genuine benefit to well‐defined populations are required to address the evidence gap.

## Author Contributions

A.A.K.L. and M.T. conducted the search and extracted the data. M.T. analysed the data. All authors made substantial contributions to the manuscript and gave final approval.

## Conflicts of Interest

Three of the five authors (M.T., L.I. and A.G.) are members of the Oxford Labour Monitoring research team at the University of Oxford, which conducts research on intrapartum fetal monitoring and decision‐support tools. This meta‐analysis was conducted independently of that work, with no funding or commercial input influencing the design, analysis, or interpretation of the findings. The other authors declare no conflicts of interest.

## Supporting information


**Table S1:** PRISMA 2020 Checklist: Guideline for Reporting Systematic Reviews and Meta‐Analyses.
**Table S2:** Outcome Measures for Inclusion in Systematic Review of Admission CTG versus IA.
**Table S3:** Details of search strategy.
**Table S4:** Participants' characteristics in studies comparing admission CTG versus intermittent auscultation.
**Table S5:** Comparison of abnormal fetal heart rate detection between admission CTG and IA across the five randomised controlled trials.
**Table S6:** Caesarean section rates among women monitored with admission CTG versus IA across five RCTs.
**Figure S1:** Forest plots for secondary outcome.

## Data Availability

The data that support the findings of this study are available from the corresponding author upon reasonable request.
