# Supplementary material for: Revisiting the Value of Admission Cardiotocography in Term Pregnancies: An Updated Systematic Review and Meta‐Analysis
Source: BJOG. 2025 Oct 11;133(3):375–90. doi: 10.1111/1471-0528.70047 (PMC12770071; doi:10.1111/1471-0528.70047)
Supplement: Supplementary file 1 — Table S1: PRISMA 2020 Checklist: Guideline for Reporting Systematic Reviews and Meta‐Analyses. Table S2: Outcome Measures for Inclusion in Systematic Review of Admission CTG versus IA. Table S3: Details of search strategy. Table S4: Participants' characteristics in studies comparing admission CTG versus intermittent auscultation. Table S5: Comparison of abnormal fetal heart rate detection between admission CTG and IA across the five randomised controlled trials. Table S6: Caesarean section rates among women monitored with admission CTG versus IA across five RCTs. Figure S1: Forest plots for secondary outcome. [file BJO-133-375-s001.docx]

**SUPPLEMENTARY MATERIAL**

Revisiting the value of admission cardiotocography in term pregnancies:

an updated systematic review and meta-analysis

**Table of contents**

[**Table S1.** PRISMA 2020 Checklist: Guideline for Reporting Systematic Reviews and Meta-Analyses. 2](#_Toc209529787)

[**Table S2**. Outcome Measures for Inclusion in Systematic Review of Admission CTG versus Intermittent Auscultation. 6](#_Toc209529788)

[**Table S3.** Details of search strategy 7](#_Toc209529789)

[**Table S4.** Participants characteristics in studies comparing admission CTG versus intermittent auscultation. 8](#_Toc209529790)

[**Table S5.** Comparison of abnormal fetal heart rate detection between admission CTG and IA across the five randomised controlled trials. 9](#_Toc209529791)

[**Table S6.** Caesarean section rates among women monitored with admission CTG versus IA across five RCTs. 10](#_Toc209529792)

[**Figure S1**. Forest plots for secondary outcomes. 11](#_Toc209529793)

# **Table S1.** PRISMA 2020 Checklist: Guideline for Reporting Systematic Reviews and Meta-Analyses.

| **Section and Topic** | **Item #** | **Checklist item** | **Location where item is reported** |
| --- | --- | --- | --- |
| **TITLE** | | |  |
| Title | 1 | Identify the report as a systematic review. | Title page |
| **ABSTRACT** | | |  |
| Abstract | 2 | See the PRISMA 2020 for Abstracts checklist. | Abstract section |
| **INTRODUCTION** | | |  |
| Rationale | 3 | Describe the rationale for the review in the context of existing knowledge. | Introduction paragraphs 1-4 describe the background of admission CTG, current practice variations, and ongoing debates about its utility. |
| Objectives | 4 | Provide an explicit statement of the objective(s) or question(s) the review addresses. | End of introduction: "We conducted an updated systematic review and meta-analysis to evaluate the impact of admission CTG on maternal and neonatal outcomes in low-risk term pregnancies." |
| **METHODS** | | |  |
| Eligibility criteria | 5 | Specify the inclusion and exclusion criteria for the review and how studies were grouped for the syntheses. | Methods section under "Eligibility Criteria" - includes study types, population characteristics, interventions, comparisons, and outcomes. |
| Information sources | 6 | Specify all databases, registers, websites, organisations, reference lists and other sources searched or consulted to identify studies. Specify the date when each source was last searched or consulted. | Methods section under "Search Strategy and Study Selection" - PubMed and Cochrane Library searched from inception to April 20, 2025. |
| Search strategy | 7 | Present the full search strategies for all databases, registers and websites, including any filters and limits used. | Methods section references Table 1 which provides detailed search terms and strategy. |
| Selection process | 8 | Specify the methods used to decide whether a study met the inclusion criteria of the review, including how many reviewers screened each record and each report retrieved, whether they worked independently, and if applicable, details of automation tools used in the process. | Methods section: "Two reviewers (M.T. and A.L.) independently screened titles and abstracts, assessed full texts for eligibility, and extracted data using a standardised form. Discrepancies were resolved through discussion." |
| Data collection process | 9 | Specify the methods used to collect data from reports, including how many reviewers collected data from each report, whether they worked independently, any processes for obtaining or confirming data from study investigators, and if applicable, details of automation tools used in the process. | Methods section: "Two reviewers (M.T. and A.L.) independently... extracted data using a standardised form. Discrepancies were resolved through discussion." |
| Data items | 10a | List and define all outcomes for which data were sought. Specify whether all results that were compatible with each outcome domain in each study were sought (e.g. for all measures, time points, analyses), and if not, the methods used to decide which results to collect. | Methods section under "Eligibility Criteria" lists all the adverse neonatal outcomes, mode of delivery, and intrapartum interventions that were collected. |
|  | 10b | List and define all other variables for which data were sought (e.g. participant and intervention characteristics, funding sources). Describe any assumptions made about any missing or unclear information. | Study characteristics extraction is mentioned in the Methods section, with details shown in Tables 2 and 7. |
| Study risk of bias assessment | 11 | Specify the methods used to assess risk of bias in the included studies, including details of the tool(s) used, how many reviewers assessed each study and whether they worked independently, and if applicable, details of automation tools used in the process. | Methods section under "Risk of bias assessment" describes using the Revised Cochrane Risk of Bias tool for randomised trials (RoB 2.0) by two independent reviewers. |
| Effect measures | 12 | Specify for each outcome the effect measure(s) (e.g. risk ratio, mean difference) used in the synthesis or presentation of results. | Methods section under "Data synthesis and statistical analysis" specifies that relative risks (RRs) with 95% confidence intervals (CIs) were calculated for dichotomous outcomes. |
| Synthesis methods | 13a | Describe the processes used to decide which studies were eligible for each synthesis (e.g. tabulating the study intervention characteristics and comparing against the planned groups for each synthesis (item #5)). | Detailed in the Methods section under "Eligibility Criteria" and "Data synthesis and statistical analysis." |
|  | 13b | Describe any methods required to prepare the data for presentation or synthesis, such as handling of missing summary statistics, or data conversions. | Methods section mentions "A continuity correction of 0.5 was applied where appropriate" for handling zero-event data. |
|  | 13c | Describe any methods used to tabulate or visually display results of individual studies and syntheses. | Forest plots are mentioned in the Methods section and shown in Figures 2 and 3. |
|  | 13d | Describe any methods used to synthesize results and provide a rationale for the choice(s). If meta-analysis was performed, describe the model(s), method(s) to identify the presence and extent of statistical heterogeneity, and software package(s) used. | Methods section: "Meta-analysis was performed using a random-effects model (DerSimonian and Laird) with inverse-variance weighting." Heterogeneity was assessed using Cochran's Q test and I² statistic. R software version 4.0.3 with metafor, meta, and metabind packages was used. |
|  | 13e | Describe any methods used to explore possible causes of heterogeneity among study results (e.g. subgroup analysis, meta-regression). | Heterogeneity assessment is mentioned with thresholds of 25%, 50%, and 75% representing low, moderate, and high heterogeneity. |
|  | 13f | Describe any sensitivity analyses conducted to assess robustness of the synthesized results. | No explicit sensitivity analyses are mentioned in the methods section. |
| Reporting bias assessment | 14 | Describe any methods used to assess risk of bias due to missing results in a synthesis (arising from reporting biases). | Methods section: "Publication bias was not formally assessed due to the limited number of studies per outcome (<10)." |
| Certainty assessment | 15 | Describe any methods used to assess certainty (or confidence) in the body of evidence for an outcome. | Methods section under "Certainty of evidence" describes using the GRADE approach to assess the certainty of evidence for each outcome. |
| **RESULTS** | | |  |
| Study selection | 16a | Describe the results of the search and selection process, from the number of records identified in the search to the number of studies included in the review, ideally using a flow diagram. | Results section first paragraph and Figure 1 (PRISMA flow diagram): "A total of 231 unique publications were identified... five RCTs met the inclusion criteria." |
|  | 16b | Cite studies that might appear to meet the inclusion criteria, but which were excluded, and explain why they were excluded. | Not explicitly mentioned in the manuscript, though the PRISMA flow diagram indicates 54 records were excluded. |
| Study characteristics | 17 | Cite each included study and present its characteristics. | Table 2 summarizes all five included RCTs with their characteristics, and they are cited in the text. |
| Risk of bias in studies | 18 | Present assessments of risk of bias for each included study. | Results section under "Risk of bias" and Table 4 presents the risk of bias assessment for each included study. |
| Results of individual studies | 19 | For all outcomes, present, for each study: (a) summary statistics for each group (where appropriate) and (b) an effect estimates and its precision (e.g. confidence/credible interval), ideally using structured tables or plots. | Forest plots in Figures 2 and 3 show individual study results with effect estimates and confidence intervals. Tables also present summary statistics. |
| Results of syntheses | 20a | For each synthesis, briefly summarise the characteristics and risk of bias among contributing studies. | Results sections on different outcomes summarize the synthesized findings and note heterogeneity or limitations where present |
|  | 20b | Present results of all statistical syntheses conducted. If meta-analysis was done, present for each the summary estimate and its precision (e.g. confidence/credible interval) and measures of statistical heterogeneity. If comparing groups, describe the direction of the effect. | Results are presented for all outcomes with relative risks, 95% CIs, and heterogeneity measures (I²) where appropriate. |
|  | 20c | Present results of all investigations of possible causes of heterogeneity among study results. | Methodological heterogeneity is discussed throughout the results section, particularly under "Admission CTG criteria and participant selection." |
|  | 20d | Present results of all sensitivity analyses conducted to assess the robustness of the synthesized results. | No explicit sensitivity analyses results are reported. |
| Reporting biases | 21 | Present assessments of risk of bias due to missing results (arising from reporting biases) for each synthesis assessed. | Not explicitly reported, though publication bias was not formally assessed as mentioned in methods. |
| Certainty of evidence | 22 | Present assessments of certainty (or confidence) in the body of evidence for each outcome assessed. | Results section under "Certainty of evidence" and Table 5 present GRADE assessments for all main outcomes. |
| **DISCUSSION** | | |  |
| Discussion | 23a | Provide a general interpretation of the results in the context of other evidence. | Discussion section "Main Findings" compares current findings with previous meta-analyses. |
|  | 23b | Discuss any limitations of the evidence included in the review. | Discussion section "Strengths and Limitations" addresses methodological heterogeneity, limited power for rare outcomes, and effects of lack of blinding. |
|  | 23c | Discuss any limitations of the review processes used. | Discussion section "Strengths and Limitations" acknowledges limitations in interpretation due to heterogeneity and evolving obstetric practices. |
|  | 23d | Discuss implications of the results for practice, policy, and future research. | Discussion sections "Clinical Relevance and Practice Implications" and "Research and Policy Implications" address these thoroughly, with five critical priorities for future research. |
| **OTHER INFORMATION** | | |  |
| Registration and protocol | 24a | Provide registration information for the review, including register name and registration number, or state that the review was not registered. | Methods section: "This systematic review and meta-analysis... registered with PROSPERO (ID: CRD420251028693)” |
|  | 24b | Indicate where the review protocol can be accessed, or state that a protocol was not prepared. | Not explicitly stated whether a separate protocol besides the PROSPERO registration was prepared. |
|  | 24c | Describe and explain any amendments to information provided at registration or in the protocol. | No protocol amendments were made. |
| Support | 25 | Describe sources of financial or non-financial support for the review, and the role of the funders or sponsors in the review. | Funding section: "This study received no specific grant from any funding agency in the public, commercial or not-for-profit sectors." |
| Competing interests | 26 | Declare any competing interests of review authors. | Conflict of interest section: The authors have no conflicts of interest to declare. |
| Availability of data, code and other materials | 27 | Report which of the following are publicly available and where they can be found template data collection forms; data extracted from included studies; data used for all analyses; analytic code; any other materials used in the review. | Not explicitly reported. |

**CTG,** cardiotocography

# **Table S2**. Outcome Measures for Inclusion in Systematic Review of Admission CTG versus Intermittent Auscultation.

| **Category** | **Specific Outcomes** |
| --- | --- |
| **Adverse neonatal outcomes** | • Apgar score <7 at 5 minutes  • Umbilical artery pH <7.05  • Neonatal intensive care unit admission  • Hypoxic–ischaemic encephalopathy  • Duration of NICU stay |
| **Mode of delivery** | • Caesarean section  • Instrumental vaginal delivery |
| **Intrapartum interventions** | • Continuous CTG use  • Fetal blood sampling  • Oxytocin use  • Epidural analgesia |
| **Other** | • Abnormal admission CTG rate |

**CTG,** cardiotocography. **NICU**, neonatal intensive care unit admission.

These selected Apgar scores and pH values were selected, because they are strongly associated with adverse neonatal outcomes (Ayres-de-Campos *et al*.,2015).

Note: Studies were eligible if they reported on at least one of these outcomes. Only studies directly comparing admission CTG with intermittent auscultation (IA) were included.

# **Table S3.** Details of search strategy

| **Search question** | “What is the potential and role of the CTG on admission for the detection of fetuses at risk for hypoxia during labour in term pregnant women presenting at the labour ward or labour assessment room with signs of labour onset?” | |
| --- | --- | --- |
| **PICO** | Population: | Term singleton pregnant women with signs of labour onset without congenital or metabolic disorders |
|  | Intervention: | Admission test (short term recording of CTG on admission) |
|  | Control: | Intermittent auscultation |
|  | Outcome: | Adverse neonatal outcomes (Apgar score < 7 at 5 minutes, umbilical cord arterial pH < 7.05, hypoxic-ischaemic encephalopathy, admission to neonatal intensive care unit), rate of obstetric interventions (caesarean section, instrumental vaginal delivery), rate of abnormal FHR in the admission test and with intermittent auscultation, rate of continuous CTG during labour |
| **Keywords** | ‘CTG’, ‘fetal monitoring’, and ‘admission’ | |
| **Search term (PubMed)**  **(Cochrane Library)** | ((cardiotocogr*[All Fields]) AND ("foetal monitoring"[All Fields] OR "fetal monitoring"[MeSH Terms] OR ("fetal"[All Fields] AND "monitoring"[All Fields]) OR "fetal monitoring"[All Fields])) AND ((admission[All Fields]) AND ("foetal monitoring"[All Fields] OR "fetal monitoring"[MeSH Terms] OR ("fetal"[All Fields] AND "monitoring"[All Fields]) OR "fetal monitoring"[All Fields]))  **Population: adults, children, pregnancy. Interventions: Admission Assessment, Cardiotocogram (CTG). Comparisons: Admission Assessment, Fetal Heart Monitoring using a Handheld Doppler, and Fetal Heart Monitoring with a Pinard Stethoscope. Outcomes: Neurodevelopmental Disorder, Vaginal Delivery, Seizure, Cerebral Hypoxia-Ischemia, Caesarean Section, Neonatal Death, Fetal Death, Continuous Fetal Heart Monitoring During Labour.** | |
| **Databases** | PubMed, Cochrane Library | |

**CTG,** cardiotocography. **FHR,** fetal heart rate.

# **Table S4.** Participants characteristics in studies comparing admission CTG versus intermittent auscultation.

| **Characteristic** | **Mires et al. (2001)^1^** | **Cheyne et al. (2003)^2^** | **Impey et al. (2003)^3^** | **Mitchell (2008)^4^** | **Smith et al. (2019)^5^** |
| --- | --- | --- | --- | --- | --- |
| **Sample size** | 3,752 | 334 | 8,580 | 582 | 3,034 |
| **Maternal age (years), mean (SD)** |  |  |  |  |  |
| • Admission CTG group | NR | 286^6^ (16–42) | 28.76 (SD 5.6) | 29.93 | 30.1 |
| • IA group | NR | 28^6^ (14–39) | 28.78 (SD 5.6) | 30 (SD 5.15) | 30.4 |
| **Nulliparous women (%)** |  |  |  |  |  |
| • Admission CTG group | NR | 44% (65) | 49% (2093) | 70% (203) | 43% (654) |
| • IA group | NR | 46% (76) | 49% (2077) | 68% (199) | 44% (668) |
| **Gestational age (weeks), mean** |  |  |  |  |  |
| • Admission CTG group | NR | NR | 40† (32–41) (2093) | NR | 39.9 |
| • IA group | NR | NR | 40† (33–41) (2077) | NR | 39.9 |
| **Birth weight (g), mean (SD)** |  |  |  |  |  |
| • Admission CTG group | NR | NR | 3590 (473) | NR | 3554 (442) |
| • IA group | NR | NR | 3602 (472) | NR | 3523 (431) |
| **Other information** |  |  |  |  |  |
| • Admission CTG group | NR | **Smoking status** Current smoker 27% (39)  Former smoker 20%  (29)  Never smoked 53% (76) | **Mean booking weight (SD)** 66.3 (11.2) kg  **Induction of labour**18% (765)  **Previous LSCS** 4% (157)  **Gestation <37 weeks** 1% (31)  **Major congenital anomaly** 1% (27) | **Ethnicity**  White: 87% (260)  Other: 11% (32)  Not stated: 2% (6) | **Labour Status on Admission***  Spontaneous labour onset: 91% (1363)  Induced labour*: 10% (149)  Not in labour caesarean section: 1% (9) |
| • IA group | NR | **Smoking status** Current smoker 33% (54)  Former smoker 19%  (31)  Never smoked 48% (77) | **Mean booking weight (SD)** 66.6 (11.5) kg  **Induction of labour** 17% (749)  **Previous LSCS** +4% (151)  **Gestation <37 weeks** 1% (32)  **Major congenital anomaly**  <1% (18) | **Ethnicity**  White: 95% (270)  Other: 5% (14)  Not stated: 0% | **Labour Status on Admission***  Spontaneous labour onset: 91% (1376)  Induced labour*: 8% (123)  Not in labour caesarean section: 1% (14) |

**Admission CTG**, Admission Cardiotocography. **IA**, Intermittent Auscultation. **NR**, Not Reported. **SD**, Standard Deviation. **LSCS**, Lower Segment Caesarean Section.

^6^Median maternal age; †Median gestational age.
Note: Smith et al. (2019) reported that 272 women presenting with signs of labour were randomized to the study, found not to be in labour, and subsequently had an induction of labour for post-dates or other clinically relevant indications.

# **Table S5.** Comparison of abnormal fetal heart rate detection between admission CTG and IA across the five randomised controlled trials.

|  | **Mires *et al.* 2001** | **Cheyne *et al.* 2003** | **Impey *et al.* 2003** | **Mitchell *et al.* 2008** | **Smith *et al.* 2019** | |
| --- | --- | --- | --- | --- | --- | --- |
| **Sample Size** | 3,752 | 334 | 8,580 | 582 | 3,034 |  |
| **Abnormal FHR on Admission** | Admission CTG: 21.5% | Admission CTG: 23% | Admission CTG 32% | NR | NR |  |
|  | IA*:  3.6% | IA:  21% | NR | NR | NR |  |

**CTG,** cardiotocography. **FHR,** fetal heart rate. *Statistically significant with a p<0.0001

# **Table S6.** Caesarean section rates among women monitored with admission CTG versus IA across five RCTs.

| **Study** | **CTG Group (%)** | | **IA Group (%)** |
| --- | --- | --- | --- |
| Impey *et al.* (2003) | 4.6 | 4.1 | |
| Mires *et al.* (2001) | 10.3 | 8.8 | |
| Cheyne *et al.* (2003) | 7.0 | 5.0 | |
| Mitchell *et al. (*2008) | 8.9 | 8.6 | |
| Smith *et al*. (2019) | 3.7 | 3.2 | |


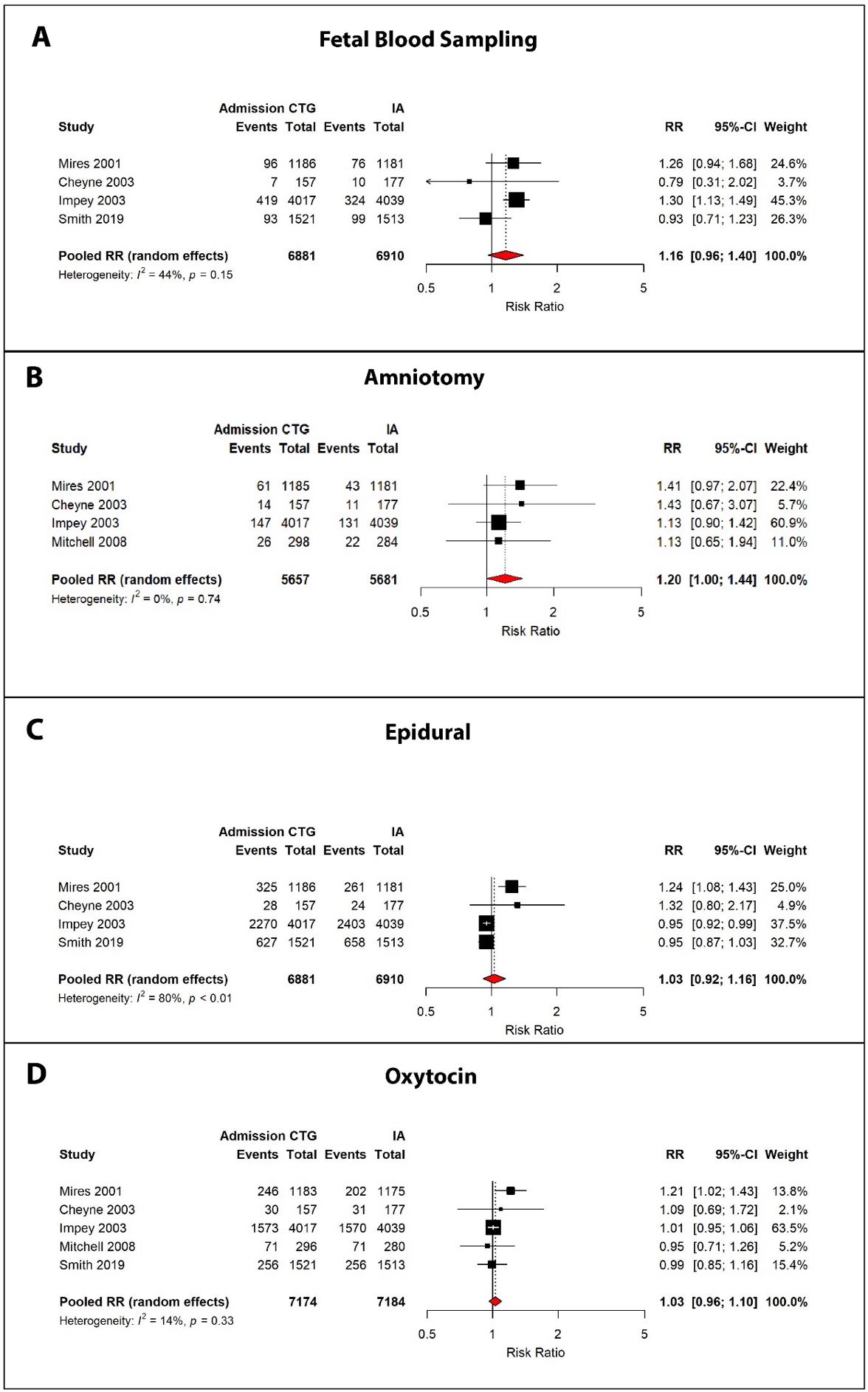


# **Figure S1**. Forest plots for secondary outcomes.
